# Effects of ketamine, s-ketamine, and MK 801 on proliferation, apoptosis, and necrosis in pancreatic cancer cells

**DOI:** 10.1186/s12871-015-0076-y

**Published:** 2015-07-29

**Authors:** Manuela Malsy, Kristina Gebhardt, Michael Gruber, Christoph Wiese, Bernhard Graf, Anika Bundscherer

**Affiliations:** Department of Anesthesiology, University of Regensburg, Franz Josef Strauss Allee 11, 93053 Regensburg, Germany

**Keywords:** Ketamine, S-ketamine, MK 801, Proliferation, Apoptosis, Necrosis, NMDA, Pancreatic carcinoma, Cancer

## Abstract

**Background:**

Adenocarcinoma of the pancreas is one of the most aggressive cancer diseases affecting the human body. The oncogenic potential of this type of cancer is mainly characterized by its extreme growth rate triggered by the activation of signaling cascades. Modern oncological treatment strategies aim at efficiently modulating specific signaling and transcriptional pathways. Recently, anti-tumoral potential has been proven for several substances that are not primarily used in cancer treatment. In some tumor entities, for example, administration of glutamate antagonists inhibits cell proliferation, cell cycle arrest, and finally cell death.

To attain endogenic proof of NMDA receptor type expression in the pancreatic cancer cell lines PaTu8988t and Panc-1 and to investigate the impact of ketamine, s-ketamine, and the NMDA receptor antagonist MK 801 on proliferation, apoptosis, and necrosis in pancreatic carcinoma.

**Methods:**

Cell proliferation was measured by means of the ELISA BrdU assay, and the apoptosis rate was analyzed by annexin V staining. Immunoblotting were also used.

**Results:**

The NMDA receptor type R2a was expressed in both pancreatic carcinoma cell lines. Furthermore, ketamine, s-ketamine, and MK 801 significantly inhibited proliferation and apoptosis.

**Conclusions:**

In this study, we showed the expression of the NMDA receptor type R2a in pancreatic cancer cells. The NMDA antagonists ketamine, s-ketamine, and MK 801 inhibited cell proliferation and cell death. Further clinical studies are warranted to identify the impact of these agents on the treatment of cancer patients.

## Background

Malignant cancer diseases are one of the major causes of death worldwide. In 2008, malignant tumors resulted in about 7.6 million deaths (approximately 13 % of all causes of death), and this figure is expected to rise to over 13 million deaths per year in 2030 [1]. One type of malignant tumor that nearly always results in death because of its aggressive behavior is adenocarcinoma of the pancreas [2]. Due to the lack of characteristic early symptoms and suitable screening tests, 50 % of patients are already affected by infiltrated lymph nodes at the time of diagnosis. According to current knowledge, this tumor stage is no longer considered curable [3]. Most of these tumors are classified as surgically incurable and have a poor prognosis for treatment [4]. Thus, the 5-year survival rate of less than 5 % is particularly unfavorable [5]. Apart from surgery, alternative and curatively successful treatment options are rare because pancreatic carcinoma is extremely resistant to radiation treatment and chemotherapy [6]. Recently, however, anti-tumoral potential has been proven in several substances that are not primarily used in cancer treatment, and these findings may open up new therapeutic options in the future [7]. Glutamate antagonists, for example, inhibit cell proliferation [8–10] and induce cell cycle arrest [11] and cell death [12] in some tumor entities. MK 801 was used for this purpose in many preclinical studies [13], but the psychotropic effects of this glutamate antagonist significantly restrict its clinical application.

Ketamine, one of the most common N-methyl-D-aspartate (NMDA) receptor antagonists used in clinical practice, was first described in the literature in 1965 and approved as an anesthetic for clinical use in 1970 [14]. Ketamine is a racemic derivative consisting of the two enantiomers s(+)ketamine and r(−)ketamine. S(+)ketamine has a four times higher binding capacity at the phencyclidine binding site of the NDMA receptor than r(−)ketamine [15]. Additionally, ketamine interacts with opioid, monaminic, and muscarinic receptors as well as with voltage-gated Ca^2+^ channels and the AMPA receptor [16]. However, the effect of ketamine fundamentally differs from that of other anesthetic drugs. Because even subnarcotic doses of ketamine result in pronounced analgesia [17], this NMDA receptor antagonist is often used in preventive pain management but also in cancer pain therapy in patients with opiate-resistant pain [18].

Aim of this study was to scan NMDA receptor type expression in pancreatic cancer cells. A further objective was to investigate the effects of ketamine, s-ketamine, and the uncompetitive NMDA receptor antagonist MK 801 on cell proliferation, apoptosis, and necrosis in pancreatic carcinoma.

## Materials and Methods

### Cell lines

The human pancreatic adenocarcinoma cell lines PaTu8988t and Panc-1 were obtained from Professor Ellenrieder (Philipps University of Marburg, Germany). PaTu8988t and Panc-1 cells were maintained in Dulbecco’s modified Eagle’s medium (Sigma-Aldrich, Steinsheim, Germany) supplemented with 10 % fetal calf serum (FCS) (Sigma-Aldrich) and 5 % Myco Zap (Lonza Verviers SPRL, Verviers, Belgium). Cells were cultured at 37 °C in humidified CO_2_ atmosphere (5 %) and maintained in monolayer culture. Experiments were done with cells at ~70-80 % confluence.

### Antibodies and reagents

Ketamine, s-ketamine, and MK 801 were purchased from Sigma-Aldrich. Final concentrations were achieved by diluting drugs in standard growth media. All solutions were prepared freshly prior to use. For immunoblotting, membranes were probed with antibodies against NMDA R1, NMDA R2a, NMDA R2b (BD Bioscience, Heidelberg, Germany), and ß-Actin (Sigma-Aldrich).

### Subcellular fractionation and immunoblotting

Cells were washed twice with cold DPBS and collected by centrifugation at 4000 rpm at 4 °C for 10 min. Lysates were then resuspended in RIPAE-Buffer (5 mL Triton X100, 190 mg EDTA, 0.5 g SDS, 2.5 g Deoxycolid Acid, 500 mL DPBS, proteinase inhibitors) for 15 min and centrifuged at 13.000 rpm for 30 min. Supernatants were transferred to new cups and incubated on ice. 30 μg of total lysates were analyzed by SDS-PAGE and blotted onto nitrocellulose. Upon protein extraction and gel transfer, membranes were washed in TBS washing buffer and incubated with peroxidase-conjugated secondary antibodies. Immunoreactive proteins were visualized by means of an enhanced chemiluminescence detection system (Western Blotting Detection Reagent, GE Healthcare).

### Cell proliferation

The quantification of cell proliferation was based on the measurement of BrdU incorporation during DNA synthesis. The test was done according to the manufactures protocol (Cell proliferation ELISA BrdU, Roche applied science, Mannheim Germany). In brief, cells were incubated with 100 μL of the test compounds for 48 h (0.1-1000 μM ketamine, s-ketamine, and MK 801). After 32 h incubation, cells were additionally treated with BrdU labeling solution for the last 16 h. The culture medium was removed, cells were fixed, and DNA was denatured. Afterwards, cells were incubated with Anti-BrdU-POD solution for 90 min, and antibody conjugates were removed in three washing cycles. Immune complexes were detected by means of TMB substrate for 15 min and quantified by measuring the absorbance at 405 nm and 490 nm. All tests were done in duplicates with eight wells per treatment group and repeated at least twice.

### Apoptosis analysis

Apoptosis assays by annexin V staining were conducted according to the manufacturer’s instructions (BD Pharming). In brief, PaTu8988t and Panc-1 cells were incubated with test reagents (10 μM ketamine, 10 μM s-ketamine, and 10 μM MK 801). Staurosporine was used for positive control and standard growth medium for negative control. After 0 h, 3 h, 6 h, 16 h, or 24 h incubation time, supernatant was decanted from the cells to preserve floating cells. Adherent cells were rinsed with warm PBS (Sigma Aldrich) and harvested by standard trypsinization. Afterwards, harvested and floating cells were mixed, washed, and resuspended in binding buffer at a final concentration of 10^6^ cells/ml. 100 μL of the cell suspension containing 10^5^ cells was resuspended in 5 μL of FITC Annexin plus 5 μL of propidium iodide, followed by 15 min incubation at room temperature protected from light. Cells were washed and resuspended with 400 μL of binding buffer. Finally, cells were analyzed by flow cytometry using FACS Calibur (BD Bioscience) and Cellquest Pro software (BD Bioscience). All tests were done in duplicates and repeated twice.

### Statistical analysis

Data are presented as mean ± SD. The non-parametric Mann–Whitney-*U*-Test was used for statistical evaluation of the data. P values < 0.05 were considered significant. IBM SPSS Statistics (Vs. 20; IBM New York, US) and Excel Vs. 2010 (Microsoft, Redmond, USA) packages were employed for statistical analysis.

## Results

### Endogenic expression of NMDA receptors in pancreatic cancer cells

**Fig. 1:**
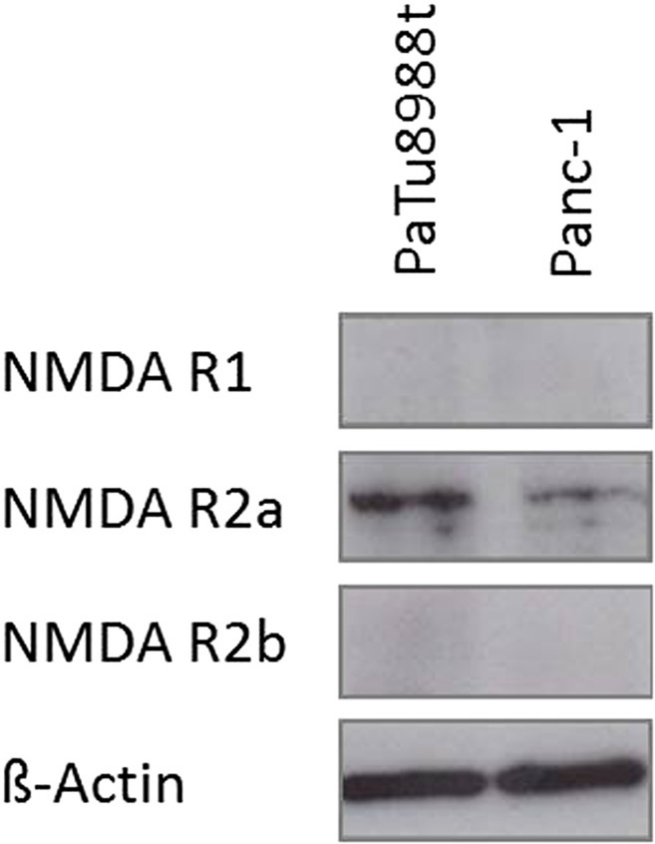
Immunblotting and proof of the endogenic expression of the NMDA receptor types R1, R2a, R2b, and ß-Actin in PaTu8988t and Panc-1 pancreatic cancer cells

The first aim was to obtain evidence for the actual expression of the NMDA receptor types R1, R2a, and R2b in pancreatic cancer cells (Fig. 1). Expression of the NMDA receptor types R1 and R2b could not be proven in any of the cell lines used (row 1 + 3). In contrast, the NMDA receptor type R2a was expressed in the pancreatic cancer cell lines PaTu8988t and Panc-1 (row 2). As control, endogenic expression of ß-Actin was shown (row 4).

### Cell proliferation behavior

**Figs. 2:**
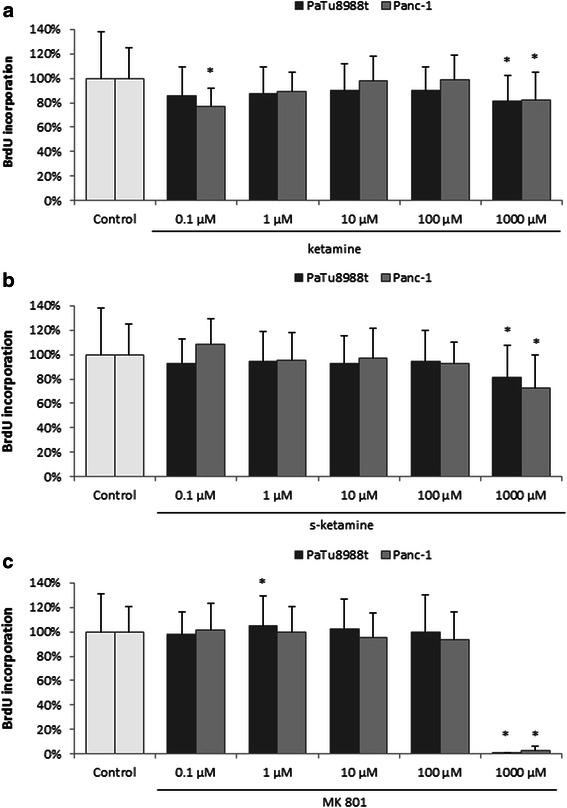
The effects of ketamine (**a**), s-ketamine (**b**), and MK 801 (**c**) on cell proliferation in PaTu8988t and Panc-1 pancreatic carcinoma cell lines in vitro. Cell proliferation was quantified by measuring BrdU incorporation. (*) indicate statistical significance at p < 0.05 compared to untreated control

PaTu8988t and Panc-1 pancreatic cancer cells were stimulated with 0.1 μM, 1 μM, 10 μM, 100 μM, and 1000 μM ketamine (a), s-ketamine (b), or MK 801 (c) each for 48 h. As a result, proliferation was significantly inhibited in both cell lines at concentrations of 1000 μM ketamine, 1000 μM s-ketamine, and 1000 μM MK 801 (Fig. 2). 1 μM MK 801 yielded a slight but significant increase in proliferation by 5 % +/− 24 % in PaTu8988t cells compared to the untreated control group (Fig. 2c). A further increase in proliferation of 23 % +/− 15 % was found in Panc-1 pancreatic cancer cells at a concentration of 0.1 μM ketamine compared to the untreated control group (Fig. 2a).

### Analysis of apoptosis and necrosis

**Figs. 3 and 4:**
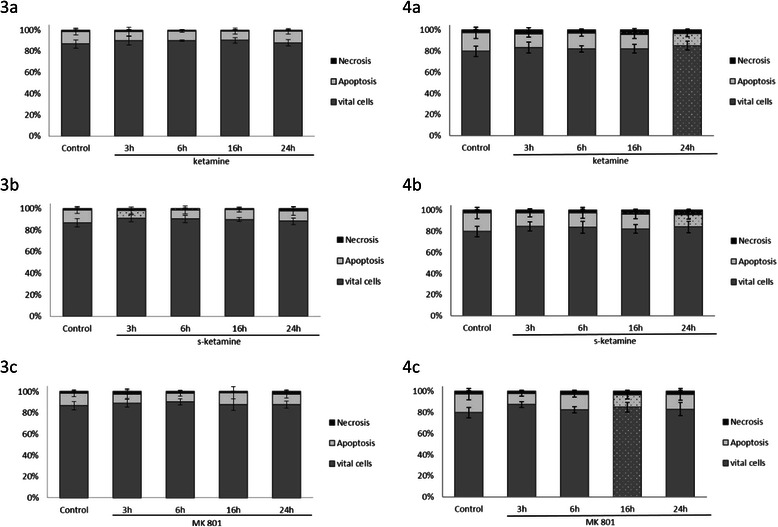
The effects of ketamine (**a**), s-ketamine (**b**), and MK 801 (**c**) on apoptosis in PaTu8988t (Figs. 3) and Panc-1 (Figs. 4) pancreatic carcinoma cell lines in vitro. For apoptosis analysis, cancer cells were stained with Annexin V. (*) indicate statistical significance at p < 0.05 compared to untreated control

The annexin V staining apoptosis assay was used to determine whether stimulation with ketamine, s-ketamine, or MK 801 incurred apoptosis or necrosis. Incubating s-ketamine in the pancreatic cancer cells PaTu8988t for 3 h (Fig. 3b) reduced the apoptotic cell fraction phase to 62 % +/− 27 % compared to the untreated samples. Ketamine and MK 801 did not induce any changes in cell death behavior.

In contrast to the untreated control samples in the cell line Panc-1, the apoptosis rate (Figs. 4) was significantly reduced after 24 h incubation with ketamine (a) (65 % +/− 17 %) and s-ketamine (b) (68 % +/− 24 %) as well as after 16 h incubation with MK 801 (c) (67 % +/− 23 %).

In contrast, the necrosis rate increased after 16 h stimulation with ketamine and, in vital cells, after 24 h incubation with ketamine (Fig. 4a).

Stimulation with s-ketamine (Fig. 4b) significantly increased necrosis after 6 h, 16 h, and 24 h, and stimulation with MK 801 (Fig. 4c) significantly increased the vital cells.

No other significant changes in the apoptosis rate were observed during the other timeframes. Staurosporine, an often employed method for inducing apoptosis, was used as a positive control for the testing procedure and induced significant apoptosis in the pancreatic cancer cells (Figure not shown).

## Discussion

Three types of ionotropic glutamate receptors are known that are termed according to their prototypic receptor-agonist N-methyl-D-aspartate (NMDA), 2-amino-3-(3-hydroxy-5-methyl-isoxazol-4-yl) propanoic acid (AMPA), and kainate (KA) [19]. N-methyl-D-aspartate (NMDA), which belongs to the family of ionotropic glutamate receptors, is vital for the transmission of stimulating signals between nerve cells [20]. Physiologically, NMDA plays a major role in processes of synaptic plasticity and memory formation [21]. NMDA is also important during various-neurolopathological conditions, such as Alzheimer disease, Parkinson disease, multiple sclerosis, epilepsy, or depression [22]. For many years, NMDA receptor expression was associated with the central nervous system, but recent trials have shown that functional NMDA receptors may also be expressed in tumors[23]. The receptor type has already been successfully proven in various tumor cell lines, for instance, in neuronal tumors (astrocytoma, glioma, and neuroblastoma), rhabdomyosarcoma, medulloblastoma, thyroid carcinoma, lung carcinoma, colon carcinoma, and mamma carcinoma as well as in T-cell chronic lymphocytic leukemia, multiple myeloma [23], and carcinoma of the larynx [25].

In the present study, NMDA receptor type R2a expression could be detected in the pancreatic carcinoma cells PaTu8988t and Panc-1. However, the influence of the NMDA receptor on the oncogenic behavior of cancer cells has not yet been sufficiently investigated. Kalariti et al. assumed in 2005 that the NMDA receptor may be a critical factor in tumor development, tumor growth, and the metastasis of tumor cells [26]. Furthermore, NMDA receptor expression in prostate carcinoma was increased in comparison to healthy prostate tissue [27]. Watanabe et al. showed in 2008 that the NMDA receptor subunit R2a plays an important role in regulating proliferation by accelerating the cell cycle in cell lines of gastric cancer [28]. In other clinical studies, blocking of the NMDA receptor subunit R2a inhibited the proliferation of cancer cells of lung carcinoma and esophagus carcinoma [29, 30].

The next step in our trial was therefore to investigate the impact of ketamine, s-ketamine, and the noncompetitive NMDA receptor antagonist MK 801 on the proliferation, apoptosis, and necrosis of pancreatic cancer cells.

In their study including 31 patients, Idyall et al. investigated the clinical effects of intravenously injected ketamine and its pharmacokinetics. After the injection of 2 mg/kg of ketamine iv, plasma concentration was analyzed by means of gas–liquid chromatography. The average maintenance dose was 41 μg/kg/min, which corresponded to a plasma concentration of 9.3 μM [31].

As our aim was to investigate the direct effect of ketamine, s-ketamine and MK 801 on cancer cell lines, we used concentrations similar to clinically achievable plasma concentrations (0,1 μM – 1000 μM).

We found that incubation with high-dose ketamine, s-ketamine, and MK 801 significantly inhibited cell proliferation in all cell lines. Lee et al. described in 2009 that administration of s-ketamine induced mitochondrial apoptosis in hepatocellular carcinoma [12]. In breast cancer cells, however, the anti-apoptotic protein BCL-2 was up-regulated, and both cell invasion and the proliferation rate were increased [32]. In our study, all test substances also decreased the rate of apoptosis in pancreatic cancer cells. The main effect of ketamine, s-ketamine, and MK 801 is based on the noncompetitive blockade of the NMDA receptor complex. During this process, ketamine binds to the phencyclidine (PCP) binding site inside the NMDA channel, inhibiting the effectiveness of NMDA antagonists, such as glutamate, NMDA, or glycine [33].

Tumor progression including the proliferation and apoptosis of cells is regulated by various signaling cascades. As a first messenger, calcium is of vital importance in this respect [34]. NMDA receptor activation increases the intracellular calcium concentration and activates Ca^2+^-dependent systolic guanylate cyclase [35]. Calcium influx into cell cytosol results in the calcium-dependent activation of a secondary messenger, which may again activate proteins of various types − amongst others transcription factors − that significantly influence the further behavior of tumor cells [36].

## Conclusions

In this study, we showed the expression of the NMDA receptor type R2a in pancreatic cancer cells. The NMDA antagonists ketamine, s-ketamine, and MK 801 inhibited cell proliferation and cell death.

The perioperative period is assumed to be a critical time with regard to cancer dissemination and metastasis [37], probably due to a fatal combination of several factors: surgical manipulation results in the release of cancer cells into circulation, growth factor levels are excessively enhanced during the wound healing process, and anticancer immune surveillance is impaired by perioperative immunosuppression [38].

Results of several preclinical and clinical studies have indicated that the selection of special anesthesia techniques, such as regional anesthesia, may affect cancer recurrence after surgery [39, 40] . Several well-established anesthetic and analgesic agents have been tested for their anticancer potency [41–45].

Modern treatment strategies of cancer diseases aim at efficaciously modulating specific signaling and transcriptional pathways (for instance, VEGF antibodies [45], tyrosine kinase inhibitors for treating chronic lymphatic leukemia [46], or EGFR antibodies for treating advanced colon carcinoma) [47]. The therapeutic potential of inhibiting Ca^2+^ channels or Ca^2+^-regulating enzymes in tumor cells has been debated for many years [48]. The basis and prerequisite of such new therapeutic approaches to a ‘targeted therapy’ necessitates comprehensive knowledge of the particular carcinogenesis. For this reason, further clinical studies are required to identify the underlying mechanisms. The identification and characterization of cellular receptors as well as their extracellular activators will help establish new therapeutic options in the treatment of aggressive pancreatic carcinoma.
